# Validation of the Ten-Item Internet Gaming Disorder Test (IGDT-10) and its association with functional impairment in Brazilian gamers

**DOI:** 10.47626/2237-6089-2023-0622

**Published:** 2024-11-06

**Authors:** Daniel Tornaim Spritzer, Wagner de Lara Machado, Marina Balem Yates, Orsolya Király, Zsolt Demetrovics, Joël Billieux, Daniel L. King, Katarzyna Kaliszewska-Czeremska, Stéphanie Laconi, Ives Cavalcante Passos, Simone Hauck

**Affiliations:** 1 Universidade Federal do Rio Grande do Sul Porto Alegre RS Brazil Programa de Pós-Graduação em Psiquiatria e Ciências do Comportamento, Universidade Federal do Rio Grande do Sul (UFRGS), Porto Alegre, RS, Brazil.; 2 Pontifícia Universidade Católica do Rio Grande do Sul Porto Alegre RS Brazil Programa de Pós-Graduação em Psicologia, Pontifícia Universidade Católica do Rio Grande do Sul, Porto Alegre, RS, Brazil.; 3 ELTE Eötvös Loránd University Institute of Psychology Budapest Hungary Institute of Psychology, ELTE Eötvös Loránd University, Budapest, Hungary.; 4 University of Gibraltar Centre of Excellence in Responsible Gaming Gibraltar Gibraltar Centre of Excellence in Responsible Gaming, University of Gibraltar, Gibraltar, Gibraltar.; 5 Flinders University College of Education, Psychology, and Social Work Adelaide Australia College of Education, Psychology, and Social Work, Flinders University, Adelaide, Australia.; 6 University of Lausanne Institute of Psychology Lausanne Switzerland Institute of Psychology, University of Lausanne, Lausanne, Switzerland.; 7 Lausanne University Hospital Center for Excessive Gambling, Addiction Medicine Lausanne Switzerland Center for Excessive Gambling, Addiction Medicine, Lausanne University Hospital, Lausanne, Switzerland.; 8 Jesuit University Ignatianum in Krakow Krakow Poland Jesuit University Ignatianum in Krakow, Krakow, Poland.; 9 Université Toulouse Jean Jaurès Toulouse France Centre d’Études et de Recherche en Psychopathologie et Psychologie de la Santé, Université Toulouse Jean Jaurès, Toulouse, France.; 10 UFRGS Hospital de Clínicas de Porto Alegre Porto Alegre RS Brazil Laboratório de Psiquiatria Molecular, Centro de Pesquisa Experimental and Centro de Pesquisa Clínica, Hospital de Clínicas de Porto Alegre (HCPA), UFRGS, Porto Alegre, RS, Brazil.; 11 UFRGS HCPA Laboratório de Pesquisa em Psiquiatria Psicodinâmica Porto Alegre RS Brazil Laboratório de Pesquisa em Psiquiatria Psicodinâmica, HCPA, UFRGS, Porto Alegre, RS, Brazil.

**Keywords:** Gaming disorder, impairment, disability, psychometrics, network analysis, Brazil

## Abstract

**Objective:**

Despite growing recognition of gaming disorder as a mental disorder, there is still debate about how best to screen for it. This is especially relevant in countries where prevalence studies that could support evidence-based policymaking have not yet been conducted. This study aims to evaluate the psychometric properties of the Brazilian Portuguese version of the Ten-Item Internet Gaming Disorder Test (IGDT-10) and to explore its association with functional impairment.

**Methods:**

An online convenience sample of 805 Brazilian adults who reported playing games completed the adapted version of the IGDT-10 and the World Health Organization Disability Assessment Schedule 2.0 (WHODAS 2.0), as well as the Problematic Internet Use Questionnaire (PIUQ), the Center for Epidemiologic Studies-Depression Scale (CES-D), the Rosenberg Self-Esteem Scale (RSES), and a socio-demographic questionnaire.

**Results:**

The Brazilian Portuguese version of the IGDT-10 demonstrated a unidimensional structure in both confirmatory and exploratory factor analyses with satisfactory internal consistency and adequate temporal stability. Participants who scored five or more on the IGDT-10 presented higher levels of functional impairment compared to those who scored positive for four symptoms or fewer. The difference between the two groups was statistically significant with a moderate effect size. Network analysis showed a direct connection between IGDT-10 scores and functional impairment and identified "negative consequences" as the most relevant item connecting these variables.

**Conclusion:**

The IGDT-10 is a brief, easy-to-understand, valid, and reliable instrument, proving to be a suitable candidate for screening gaming disorder in future epidemiological studies.

## Introduction

Gaming is one of the main leisure activities for children, adolescents, and adults and it is estimated that over 3 billion people around the world play video games.^1,2^ In Brazil, the leading game market in Latin America, approximately 75% of the Brazilian population play video games between the ages of 16 and 24 years.^3^ Although gaming is healthy and beneficial for the vast majority, approximately 2% of the world population may experience significant negative consequences resulting from a persistent pattern of uncontrolled, prioritized, and continued gaming behavior.^4^ Gaming disorder (GD) is more common in adolescents and young adults than in children and older adults; it affects more boys than girls and it is associated with a number of psychological and psychiatric conditions.^5,6^ The evidence that GD had a global public health impact led first to its consideration as a tentative disorder in section 3 of the Diagnostic and Statistical Manual of Mental Disorders, 5th edition (DSM-5^7^) (remaining unchanged in DSM-5-TR) and later to its inclusion as an official diagnosis (6C51) in the International Classification of Diseases-11 (ICD-11).^8–11^ Despite growing recognition of GD as a mental disorder, there is still debate about how to best screen for it and assess it. This is especially relevant in countries like Brazil, where prevalence studies that could support evidence-based policymaking have not yet been conducted.^12^ It is thus important that future prevalence studies in the country can benefit from the availability of a validated and psychometrically robust instrument to screen for GD in the general population.

The Ten-Item Internet Gaming Disorder Test (IGDT-10)^13^ is considered one of the most valid and reliable tools for screening for GD symptoms.^14,15^ The IGDT-10 stands out as a brief self-report screening instrument that uses simple, clear, and consistent item wording that adequately reflects the GD concept.^13^ These features are essential for its use in population-based surveys, particularly in developing countries, where education outcomes tend to vary significantly depending on socioeconomic background.^16^ This measurement instrument covers all of the DSM-5 criteria and items from the IGDT-10 can also be used to approximate the proposed GD clinical guidelines included in the ICD-11.^17,18^ Unlike most instruments developed after the DSM-5, the IGDT-10 investigates GD-related negative consequences via two separate items. Given the complexity of DSM-5 criterion 9 for GD ("Has jeopardized or lost a significant relationship, job, or educational or career opportunity because of participation in Internet games"), Király et al.^13^ operationalized it with two items to facilitate understanding and to avoid relying on a double-barreled question. But since these items refer to the same DSM-5 criterion, they are later combined for analysis (a positive response to either or both of these items only adds one point to the final score). Previous validation studies have shown that the IGDT-10 has a single factor structure, satisfactory internal consistency, and adequate construct and criterion validity.^11,19^ It has been validated in a large number of Western and Eastern countries, and its gender and language invariance has been tested in a large international sample with more than 7,000 gamers.^19–22^

Identification of functional impairment plays a central role in assessment of GD. This helps to differentiate between intensive but healthy and pathological involvement in video games, reducing the risk of over-pathologizing gaming patterns.^23,24^ It also prevents prevalence overestimation in epidemiological studies,^4^ and allows more accurate detection of the clinical and neurobiological correlates associated with GD.^25–27^ From a psychometric and psychopathological point of view, it is also useful to understand which items of an instrument are most related to functional impairment. Added to this is the criticism that instruments developed from DSM-5 (IGDT-10 included) follow the manual's nonhierarchical approach, in which cases at risk of GD are identified based on any five out of the nine criteria, even without endorsement of "negative consequences."^28^ Ko et al.^18^ found that DSM-5 criterion 9 was the item that best distinguished between gamers with and without GD, with 94.7% diagnostic accuracy when compared to psychiatric interviews. Lee et al.^29^ identified that this item was more frequent in gamers with severe GD, suggesting that this criterion should have a higher hierarchic order among DSM-5 criteria. More recently, Castro-Calvo et al.^30^ investigated experts’ appraisals of GD criteria using Delphi methodology and suggested that DSM-5 criterion 9 provided the highest diagnostic validity, clinical utility, and prognostic value of all the DSM-5 criteria.

### The present study

This study aimed primarily to assess the psychometric properties of the Brazilian version of the IGDT-10. Based on previous studies, we hypothesized that the Brazilian version of the IGDT-10 would demonstrate a unidimensional factor structure, with good internal consistency and satisfactory temporal stability. Along with exploring the construct validity of the IGDT-10 in relation to demographic, gaming, and psychopathological variables, to further assess the instrument's clinical relevance, we also used a standard instrument, the World Health Organization Disability Assessment Schedule 2.0 (WHODAS 2.0), to investigate the association between IGDT-10 scores and functional impairment. We expected that participants who scored above the IGDT-10 cutoff point would have higher levels of disability than those who scored below this threshold. In exploring the role of specific IGDT-10 items in the association with functional impairment, we hypothesized that the "negative consequences" criterion may play a prominent role.

## Methods

This is a cross-sectional study that is part of the multicentric project titled "Cross-cultural Internet and mobile phone uses," conducted in 14 countries, from September 2018 to July 2019. Since this component of the project also aimed to assess the relationship between problem gaming and functional impairment, the Brazilian protocol includes both the IGDT-10 and the WHODAS 2.0.

### Participants and procedures

A convenience sample of Brazilian adults (≥ 18 years) who reported playing games was recruited online via social media platforms (especially Facebook and WhatsApp) and e-mail, from September 2018 to July 2019. We estimated a sample size of between 500 and 1,000 participants, which is considered adequate for carrying out the confirmatory factor analysis (CFA) and other psychometric tests.^31^

Data were collected anonymously using SurveyMonkey^®^ and no identifying information (e.g., internet protocol addresses) was collected. At the end of the questionnaire, participants were offered feedback on problematic gaming, internet, and smartphone use, for which an e-mail address was requested. In August 2019, those who provided an e-mail address were invited to answer the IGDT-10 scale a second time to enable test-retest validation. To ensure confidentiality, the feedback e-mail containing the invitation to participate in the retest was sent in an automated manner using Mail Merge for Gmail^®^, so that researchers did not have access to participants’ questionnaire scores and e-mail addresses simultaneously. The interval between the test and retest was at least 4 weeks.

### Measures

#### Sociodemographic and gaming use data

Participants were asked about their age, sex, education, employment, and marital status, as well as the number of hours of daily gaming and the main platform used for gaming (computers, consoles, smartphones, or tablets). Self-perception of problematic gaming was assessed with the question "Over the past year, do you feel that you have had problematic gaming use?" Participants were provided with a four-point Likert scale of "no," "rather no," "rather yes," and "yes."

#### IGDT-10

This questionnaire assesses GD in recent years with 10 items that address the nine diagnostic criteria for internet GD proposed in the DSM-5.^13^ Each criterion was operationalized using a single item, except for criterion 9, referring to "jeopardized or lost a significant relationship, job, or educational or career opportunity because of participation in internet games," which was assessed with two separate items. All questions have Likert-type responses ranging from 0 (never), 1 (sometimes), to 2 (often). However, to maintain similarity with the dichotomous approach used by the DSM-5, "never" and "sometimes" responses are coded as not meeting the criterion (0 points), while "often" is coded as meeting the criterion (1 point). Items 9 and 10 refer to the same DSM-5 criterion and are combined for analysis. Answering "often" for either or both of these items adds just one point to the final score. Thus, the IGDT-10 score ranges from 0 to 9, and a score of 5 or more points (IGDT-10 problematic status) identifies individuals at risk of GD according to the DSM-5. Since there is an established cutoff point, we analyzed the IGDT-10 as a categorical variable.^13,19,22^ Previous validation studies have shown that the IGDT-10 has a one-factor structure.^11,17–19^ Considering nine items and dichotomous answers, the internal consistency measured by Cronbach's alpha ranged from 0.68 to 0.79.^13,21^

Cultural adaptation of the IGDT-10 for Brazilian Portuguese followed well-established cross-cultural adaptation guidelines,^32^ consisting of forward translation, back-translation, expert committee review, and face validity evaluation. The instructions, items, and response options of the English version of the IGDT-10 were forward translated independently by two groups of three bilingual mental health professionals whose native language was Brazilian Portuguese, producing two Brazilian Portuguese versions. An expert committee comprising 15 members skilled in psychometric research and in internet use disorders examined both translated versions to assess linguistic and semantic discrepancies and developed a synthesized translation version by consensus. Two back-translations were then produced independently by two native English speakers who have lived in Brazil for many years, one being a psychologist born in the United States and the other an English teacher born in England. They were not informed of the objectives of the study and had no previous knowledge about the questionnaire being adapted. These versions were then evaluated to check how much they differed from the original instrument in terms of meaning, using a four-point Likert scale from 1 (greatly altered) to 4 (not altered). At a second meeting of the expert committee, the items were revised based on the insights from the back-translations and, when necessary, consensually adjusted to maintain the meaning of the original instrument, producing a new synthesized and unified version in Brazilian Portuguese. Face validity was evaluated by 15 people who were asked for comments and suggestions regarding the clarity and comprehensibility of each item and the whole questionnaire.

#### The WHODAS 2.0

WHODAS 2.0 is a reliable and valid measure of health and functional impairment/disability. It comprises 12 items that assess six different dimensions: cognitive functions, mobility, self-care, getting along, life activity, and participation. Answers to the questions are classified on a five-point Likert-type scale indicating the level of difficulty or problem, from 0 (none) to 4 (extreme difficulty or inability to perform). Scores were computed using the official item response theory-based WHODAS recommendation.^33^ Each item response is treated separately, and the summary score is generated by differentially weighting the items and the levels of severity. The steps to compute the score include summing the recoded item scores within each domain, summing all six domain scores, and then converting the summary score into a metric score ranging from 0 to 100 (where 0 = no disability; 100 = full disability). The WHODAS 2.0 Brazilian cultural adaptation was approved by World Health Organization (WHO). Based on a study conducted in 36 countries, the WHODAS 2.0 has high internal consistency as measured by Cronbach's alpha (*α* = 0.86) and other psychometric properties of this instrument are also considered to be very good.^33^

#### Problematic Internet Use Questionnaire – Short Form-9 (PIUQ-SF-9)

The PIUQ-SF-9 consists of nine items that evaluate problematic internet use (PIU) according to three dimensions: obsession, neglect, and control disorder.^34^ All items are rated on a five-point Likert-type scale, ranging from 1 (never) to 5 (almost always/always). Total scores range from 9 to 45, and higher scores indicate a higher risk of PIU. The PIUQ-SF-9 has demonstrated high internal consistency across different studies, with Cronbach's *α* values ranging from 0.81 to 0.93.^34–36^

#### Center for Epidemiologic Studies - Depression Scale-10 (CES-D-10)

This is a brief version of the CES-D designed to assess depressive symptoms. It consists of 10 items that are evaluated on a Likert-type scale ranging from 0 (rarely or never) to 3 (most of the time or all the time). Scores can range from 0 to 30, and a cutoff of 10 or more is indicative of significant depressive symptomatology.^37^ Cronbach's *α* was higher than 0.80 in all subgroups in both the original study and a Brazilian validation study.^38,39^

#### Rosenberg Self-Esteem Scale (RSES)

The RSES consists of 10 items assessed on a four-point Likert-type scale ranging from 1 (strongly disagree) to 4 (strongly agree). The scale comprises five positive and five negative statements, and the negative items are reverse scored before analysis. The scale ranges from 10 to 40. Scores between 25 and 35 are considered to be within the normal range, while scores below 25 suggest low self-esteem.^40^ Reliability of the Brazilian version of RSES measured by Cronbach's *α* ranges from 0.70 to 0.90.^41,42^

### Data analysis

Analyses were performed using R (version 3.2.2) implemented with the following packages: scales (v.1.1.1),^43^ car (v3.0-10),^44^ psych (v2.1.3),^45^ lavaan (v0.6-9),^46^ semTools (v0.5-3),^47^ qgraph (v1.6.9),^48^ IsingFit (v0.3.1),^49^ and bootnet (v1.4.3).^50^ All participants who filled in the sociodemographic data and completed the IGDT-10 were included. No imputation or replacement techniques were used to handle missing data; estimations were made using pairwise information.

#### Factor structure and reliability

The internal structure of the IGDT-10 was assessed in several analyses. The factorability of sample data was assessed using Bartlett's test of sphericity and the Kaiser-Meyer-Olkin (KMO) index. First, as this is the first IGDT-10 study in a Brazilian setting, we conducted an exploratory factor analysis (EFA) with oblique rotation and a parallel analysis retention method to identify the latent variables of the IGDT-10.^51,52^ Second, we performed a CFA to verify the structural validity of the instrument, considering the following fit indices to indicate the model's adequacy: comparative fit index (CFI) and Tucker-Lewis index (TLI) ≥ 0.95, root mean square error of approximation (RMSEA) ≤ 0.06, with associated p-value and standardized root mean residual (SRMR) ≤ 0.10.^53^ We considered structural coefficient loadings according to Comrey and Lee's recommendations,^54^ which were based on the percent of the variable's variance in common with the factor. They considered that loadings ≥ 0.71 = excellent, > 0.63 = very good, > 0.55 = good, > 0.45 = fair, and > 0.32 = poor. Both EFA and CFA were performed using the same total sample. As there was a low number of extreme cases, we opted not to stratify the sample to avoid decreasing the power of the analysis.^55^

Internal consistency of the IGDT-10 was assessed considering the final nine items with dichotomous answers using McDonald's asymptotic hierarchical omega coefficient (ωH), which is considered satisfactory if higher than 0.70.^56,57^ Cronbach's alpha (α) is also reported for the sake of comparability with previous research. The intraclass correlation coefficient (ICC) and corresponding 95% confidence interval (95%CI) were calculated to estimate test-retest reliability, which is considered adequate for values between 0.50 and 0.75, good for values between 0.75 and 0.90, and excellent for values > 0.90.^58^

#### Construct validity

Bivariate and partial correlation analyses were conducted to evaluate how IGDT-10 problematic status (scoring ≥ 5) correlated with sex, age, time spent gaming, self-perception of problematic gaming, PIU, self-esteem, depression symptoms, and functional impairment. For both analyses, instruments with a well-established cutoff point (IGDT-10, CES-D-10, and RSES) were treated as categorical variables, while the remainder (PIUQ-SF-9 and WHODAS 2.0) were evaluated as continuous variables.

To assess the relationship between risk of GD and functional impairment, we used the Mann-Whitney *U* test to estimate a rank biserial correlation (and its significance and effect size) between IGDT-10 problematic status and the WHODAS 2.0 total score. A linear regression analysis was also implemented to evaluate the impact on functional impairment associated with (a) each one-point increase in the IGDT-10 score, and (b) IGDT-10 problematic status.

We also developed two network models to further explore the construct validity of the IGDT-10 considering its association with WHODAS 2.0. The first was a nomological network designed to explore the relationship between IGDT-10 problematic status and functional impairment considering the influence of other variables: sex, age, time spent gaming, self-perception of problematic gaming, PIU, self-esteem, and depression symptoms. Here, the nodes represent the variables, and the edges represent their partial correlations (or partial linear regression coefficients). These correlations can be positive (blue edges) or negative (red edges), and the greater the strength of the correlation, the thicker the edge.^59^ In the second network, we aimed to illustrate how the relationship between IGDT-10 items and disability occurred at the symptom level, that is, which IGDT-10 symptoms had a direct connection with WHODAS 2.0. For this purpose, the node representing IGDT-10 problematic status in the previous network was replaced by nine nodes representing specific IGDT-10 symptoms. The accuracy and stability of centrality measures were assessed by sample permutation bootstrapping (n = 500 resamples).^60,61^ Accuracy of edge weights and centrality measures was estimated with the 95%CI of bootstrapped samples (n = 500) while stability of centrality was estimated by case-dropping correlation with original estimates (from 95 to 25% of cases).

Data availability

The dataset and the syntax of the analysis presented in this study are fully available online in the Open Science Framework (OSF) repository at https://osf.io/wcjn5/

### Ethical considerations

This study was performed in line with the principles of the Declaration of Helsinki. Approval was granted by the research ethics committee at the Hospital de Clínicas de Porto Alegre (protocol number 89702318.2.0000.5327). Informed consent, including consent for publication, was obtained from all individual participants included in the study.

## Results

### Cultural adaptation

The two forward translations achieved comparable results and only minor adjustments were needed to produce the first synthesized version. In general, refinements suggested by the experts aimed to simplify the language of the questionnaire and make it more colloquial, considering its use in adolescent populations as well (even though this specific study was conducted with adults). In the back-translation, items maintained their meaning compared with the original instrument. Regarding face validity, the questionnaire was rated as "easy to understand" by all the respondents in a pre-test group of 15 people. The final Brazilian Portuguese version of the IGDT-10 is available in Supplementary Material S1.

### Demographic data

The final sample consisted of n = 805 participants. The majority were female (n = 530, 65.8%), and the mean age was 36.0 ± 13.0 years (age range 18-72). Of these, n = 124 responded to the IGDT-10 retest at an average of 6 months after the first completion. The majority were female (n = 88, 70.1%) and the mean age was 34.1 ± 13.0 years (age range 18-71). The main sociodemographic data of the test and retest samples are presented in Table 1.

**Table 1:** Descriptive statistics of sociodemographic variables

		Entire sample (n = 805)	Retest sample (n = 126)
Mean age in years (SD)		36.00 (12.96)	34.11 (12.98)
		
Gender			
	Women	530 (65.8)	88 (69.8)
		
Occupation, n (%)			
	Studying only	150 (18.6)	34 (27.0)
	Studying and working	216 (26.8)	37 (29.4)
	Working only	376 (46.7)	42 (33.3)
	Not working, not studying	63 (7.8)	13 (10.3)
		
Educational level, n (%)			
	High school, incomplete	9 (1.1)	-
	Elementary school	14 (1.7)	-
	High school, complete	46 (5.7)	7 (5.6)
	High school, complete + 1-3 years of study	105 (13.1)	24 (19.0)
	High school, complete + 4-6 years of study	175 (21.7)	28 (22.2)
	High school, complete + 7 or more years of study	453 (56.3)	66 (52.4)
		
Marital status, n (%)			
	Single	231 (28.7)	42 (33.3)
	Dating	129 (16.0)	27 (21.4)
	Living together	132 (16.4)	13 (10.3)
	Married	253 (31.4)	31 (24.6)
	Divorced	53 (6.5)	11 (8.7)
	Widowed	6 (0.7)	2 (1.6)

SD = standard deviation.

### GD prevalence and criteria endorsement

Among all participants, 75.9% (n = 611) did not respond positively to any of the criteria. "Escape" was the most frequently endorsed criterion (13.7%) in this sample, followed by "continuation" (8.2%) and "preoccupation" (7.9%). Based on the cutoff point of ≥ 5 criteria, 3% (n = 24) of the sample were considered at risk for GD. Among these, "escape" was the most endorsed criterion (91.6%), followed by "tolerance" (87.5%) and "giving up other activities" (85.7%) (Table 2).

**Table 2:** IGDT-10 pattern coefficients and item endorsement

Items	EFA	CFA	Item endorsement among all gamers (n = 805) n (%)	Item endorsement among problem gamers (n = 24) n (%)
1. Preoccupation	0.829	0.834	63 (7.9)	18 (75.0)
2. Withdrawal	0.849	0.859	33 (4.1)	16 (66.6)
3. Tolerance	0.908	0.907	52 (6.5)	21 (87.5)
4. Loss of control	0.794	0.759	34 (4.3)	13 (54.1)
5. Giving up other activities	0.885	0.883	32 (4.0)	18 (85.7)
6. Continuation	0.868	0.854	65 (8.2)	20 (83.3)
7. Deception	0.841	0.836	27 (3.4)	13 (54.1)
8. Escape	0.828	0.808	109 (13.7)	22 (91.6)
9. Negative consequences	0.879	0.865	15 (1.9)	10 (41.6)

CFA = confirmatory factor analysis; EFA = exploratory factor analysis; IGDT-10 = Ten-Item Internet Gaming Disorder Test.

### Factor structure and reliability

Bartlett's test of sphericity (χ^2^[36] = 1896.31, p < 0.001) and the KMO (0.87) measure of sampling adequacy indicated that the data were appropriate for factor analysis. In the EFA, the unidimensional model accounted for 72.9% of the common variance of the items. The CFA model indicated an optimal fit to the data (χ^2^ = 38.444, degrees of freedom [df] = 27, CFI = 0.995, TLI = 0.993, RMSEA = 0.023 [0.000-0.039], RMSEA p close = 0.999 and SRMR = 0.055). All items had excellent loadings in a single-factor structure (Table 2).

Regarding the internal consistency of the IGDT-10, ωH was 0.84 and *α* was 0.95. For the test-retest reliability, the ICC was 0.59 (95%CI 0.49-0.68).

### Construct validity

The results of the bivariate and partial correlation analysis of IGDT-10 problematic status and sex, age, time spent gaming, self-perception of problematic gaming, PIU, self-esteem, depression symptoms, and functional impairment are presented in Table 3.

**Table 3:** Heatmap of bivariate and regularized partial correlations among the risk of gaming disorder (scoring 5 or more on IGDT-10), functional impairment, sex, age, time spent gaming, self-perception of problematic gaming, problematic internet use, self-esteem, and depression symptoms

	Sex	Age	TSG	SPP	RSES	CES-D-10	PIUQ-SF-9	IGDT-10	WHODAS 2.0
Sex	-	-0.22	0.21	0.17	0.04	-0.07	-0.03	0.32	-0.03
Age	-0.19	-	-0.21	-0.02	-0.51	-0.27	-0.29	-0.31	-0.21
TSG	0.10	-0.10	-	0.47	0.25	0.21	0.22	0.38	0.13
SPP	0.00	0.21	0.33	-	0.12	0.18	0.34	0.61	0.16
RSES	-0.09	-0.34	0.08	-0.15	-	0.49	0.32	0.37	0.39
CES-D-10	-0.01	-0.06	0.07	0.20	0.36	-	0.41	0.10	0.48
PIUQ-SF-9	-0.17	-0.13	0.00	0.07	-0.04	0.24	-	0.46	0.43
IGDT-10	0.25	-0.14	0.03	0.53	0.27	-0.33	0.27	-	0.34
WHODAS 2.0	-0.06	0.04	-0.03	-0.10	0.09	0.33	0.17	0.22	-

Age = older age; CES-D-10 = Center for Epidemiologic Studies - Depression Scale-10; IGDT-10 = Ten-Item Internet Gaming Disorder Test; PIUQ-SF-9 = Problematic Internet Use Questionnaire *–* Short Form-9; RSES = Rosenberg Self-Esteem Scale; Sex = male sex; SPP = self-perception of problem gaming; TSG = time spent gaming; WHODAS 2.0 = World Health Organization Disability Assessment Schedule 2.0. Bivariate correlation analyses are presented in the upper diagonal while regularized partial correlation analyses are presented in the lower diagonal. Blue color indicates a positive correlation between variables, while red color indicates a negative correlation. The stronger the correlation, the more intense the color. For both analyses, instruments with a well stablished cutoff point (IGDT-10, CES-D-10 and RSES) were treated as nominal variables, while the remainder (PIUQ-SF-9 and WHODAS 2.0) were evaluated as continuous variables.

Participants who answered positively to five or more symptoms on the IGDT-10 presented higher levels of functional impairment (mean = 31.99, standard deviation [SD] = 20.07, median = 31.58) measured by the WHODAS 2.0 total score when compared to those who endorsed a positive response to up to four symptoms (mean =15.57, SD = 14.19, median = 10.53). The difference between the two groups was statistically significant (U = 2940.5; p < 0.001) and showed a moderate effect size (r_pb_ = 0.34).

The linear regression model showed that each one-point increase in the IGDT-10 score was associated with a 2.88 increase in the WHODAS 2.0 score and endorsing a positive response to five or more symptoms was associated with a 16.43 increase in the WHODAS 2.0 score.

**Figure 1:**
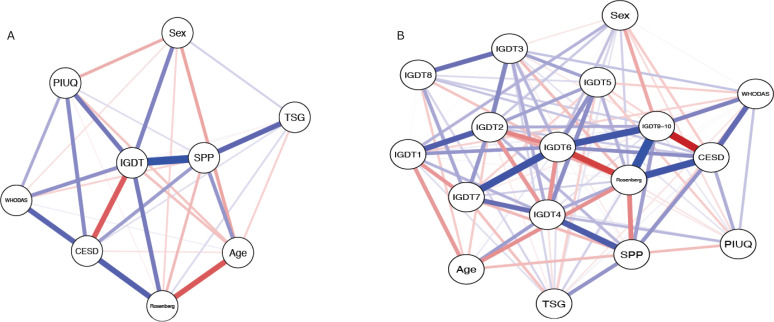
Network analysis of Ten-Item Internet Gaming Disorder Test (IGDT-10) and its association with functional impairment, considering both its problematic status and specific symptoms. A) IGDT-10 problematic status’ network. B) IGDT-10 specific symptoms’ network. Age = younger age; CES-D = depressive symptoms (Center for Epidemiologic Studies - Depression Scale-10 [CES-D-10]); IGDT = gaming disorder (5+ on IGDT-10); IGDT1 = preoccupation; IGDT2 = withdrawal; IGDT3 = tolerance; IGDT4 = loss of control; IGDT5 = giving up other activities; IGDT6 = continuation; IGDT7 = deception; IGDT8 = escape; IGDT9-10 = negative consequences; PIUQ = Problematic Internet Use Questionnaire – Short Form-9 (PIUQ-SF-9); Rosenberg = Rosenberg Self-Esteem Scale (RSES) (lower); Sex = male sex; SPP = self-perception of problem gaming; TSG = time spent gaming; WHODAS = functional impairment (World Health Organization Disability Assessment Schedule 2.0 [WHODAS 2.0]). Nodes represent the variables, and the edges represent their partial correlations (or partial linear regression coefficients). These correlations can be positive (blue edges) or negative (red edges) and the greater the strength of the correlation, the thicker the edge. Edge accuracy, edge stability, and stability of centrality measures by the bootstrap method of the network models are presented in Supplementary Material S2.

In the nomological network (Figure 1A), IGDT-10 problematic status showed a direct connection with functional impairment. In addition, two indirect connections between these variables were also identified: one associated with PIU and the other associated with low self-esteem and depression. Additionally, IGDT-10 problematic status and self-perception of problem gaming are strongly connected and present higher expected influence levels. The symptom level network (Figure 1B) showed that "negative consequences" was the node that was most strongly connected with functional impairment. This association followed the same pattern observed in the previous network. There was a direct connection between "negative consequences" and functional impairment and two indirect connections: one via PIU and another via self-esteem and depression. Additionally, "loss of control" and "negative consequences" were the variables with the highest expected influence in this network, followed by "tolerance" and "continuation." Edge accuracy, edge stability, and centrality stability measures by the bootstrap method of the network models are presented in Supplementary Material S2.

## Discussion

The present study found that the Brazilian version of the IGDT-10 has solid psychometric properties, including (a) unidimensional factor structure, (b) satisfactory internal consistency and adequate test-retest reliability, and (c) construct validity, demonstrated by the associations with demographic, gaming, psychopathological variables, and functional impairment. This study also contributes to the field by being the first to examine the temporal stability of the IGDT-10 and to explore its construct validity using a standard functional impairment measure such as the WHODAS 2.0.

The unidimensional factor structure of the IGDT-10 was demonstrated using multiple techniques. The exploratory analysis suggested retention of one factor, and the confirmatory approach presented optimal fit indexes for this single factor solution. These findings are in line with previous psychometric research and have already been demonstrated by both EFA^21^ and CFA^13,19,20^ approaches. To date, no validation studies have evaluated the factor structure of the IGDT-10 using both EFA and CFA conducted on independent subsamples within the same study.^14^

We assessed the reliability of the IGDT-10 in terms of internal consistency and temporal stability. Internal consistency was measured using both McDonald's asymptotic omega and Cronbach's alpha considering the IGDT-10's nine variables in the binary format and was found to be quite satisfactory. This is in line with previous psychometric studies, although the alpha value in our study was slightly higher. However, we used Cronbach's alpha exclusively to facilitate comparison with previous data, since McDonald's omega had not previously been used to measure the internal consistency of the IGDT-10. We favor McDonald's omega because it is more appropriate in situations where the variance of items composing a scale is not necessarily comparable, which is especially true in psychological research.^56^ This is the first study showing that the stability of IGDT-10 is adequate, although at a lower magnitude compared to other representative GD scales, such as the IGDS9-SF, GAS-7, or Lemmens IDG-9.^62^ This may partly be explained by the extended time elapsed before retesting, which occurred on average 6 months after the first administration. Therefore, some changes in symptomatology can be expected, particularly considering that we relied on a nonclinical sample susceptible to experiencing contextual variation in their gaming patterns.

Considering demographic, gaming, and psychopathological measures,^63^ IGDT-10 problematic status correlated with male sex, younger age, time spent gaming, self-perception of one's gaming pattern as problematic, PIU, and lower self-esteem, which is in alignment with previous studies.^11,17–19^ These findings reinforce that GD is a multifaceted phenomenon, resulting from a complex interaction between intrinsic factors (intra and interpersonal) and extrinsic factors (social and technological).^63^ Considering the demographic characteristics of participants who scored positive for IGDT-10 problematic status, educational initiatives could be targeted towards young boys who engage in daily gaming for extended periods and who also use the internet excessively for activities other than gaming. From a clinical perspective, careful evaluation of self-esteem should be a central aspect in investigating GD, allowing for a more comprehensive diagnostic understanding and development of a treatment plan that is both more specific and effective. One unexpected finding of our study, however, was the correlation with depressive symptoms, which turned out to be very weak (0.10) in the bivariate analysis and moderate but negative (-0.33) in the multivariate analysis. One possible explanation for this finding can be raised through the network analysis, which shows that low self-esteem acts as a bridge between depressive symptoms and GD. In partial correlations analysis, when two out of three variables show a positive correlation, a third spurious negative correlation can emerge as a residual of what is not shared by the other variables.^60^

Functional impairment related to GD may be personal (sleep disturbances, basic hygiene neglect), social (isolation, conflicts with friends and family), educational (loss of interest, missed educational opportunities, school dropout), professional (reduced productivity, loss of employment), or financial (overspending).^64,65^ Identification of impairment is one of the essential features for a diagnosis of GD, given its role in distinguishing individuals with GD from the significant proportion of those engaging in intense gaming patterns without experiencing negative consequences.^24^

In our study, participants with IGDT-10 problematic status also presented higher levels of functional impairment assessed by WHODAS 2.0, and this association was statistically significant and had a moderate effect size. The mean and median WHODAS 2.0 score of participants who endorsed a positive response for five or more IGDT-10 symptoms was equivalent to the 95th percentile of the general population, considering the normative functional impairment data for the adult population worldwide.^33^ Previously, Pearcy et al.^66^ employed the WHODAS 2.0 to assess functional impairment associated with GD in the validation study of the PIE-9. Bivariate analysis showed that individuals at risk of GD according to the PIE-9 had significantly higher levels of disability than individuals who scored below the instrument's cutoff point. Based on normative data for the Australian population,^67^ the mean WHODAS 2.0 score in the group at high risk for GD was equivalent to the 95th percentile of the general population, while the mean score of the group at low risk was equivalent to the 85th percentile.

Exploring the construct validity of the IGDT-10 through its association with functional impairment, two findings from the network analysis are worth noting. The first is the demonstration of a direct relationship between IGDT-10 problematic status and functional impairment, which shows itself independent of other factors, such as depressive symptoms. This is important because the WHODAS 2.0 is an instrument that assesses functioning and functional impairment generically and is not disorder-specific, potentially raising questions about whether the impairment is due to the gaming behavior or, for example, to associated comorbidity. The second is recognizing that the connection between the IGDT-10 and functional impairment at the symptom level occurs via the "negative consequences" symptom. This finding is in line with previous studies that have already highlighted this symptom's diagnostic validity, clinical utility, and prognostic value.^18,29,30^ The finding that "negative consequences" plays a pivotal role in maintenance of GD also supports the view that it should be assessed in a straightforward manner and with plain language, as done using the IGDT-10.

### Limitations and future directions

Some limitations should be considered when interpreting the results of this study. First, our sample was not recruited using probabilistic procedures, which may hinder the generalization of these findings to the general population or even to a population of gamers. Second, all information was gathered using self-report questionnaires, which can introduce, for example, social desirability and short-term recall biases. Third, since scoring specific functional impairment dimensions in the 12-item version of the WHODAS 2.0 is not recommended, we did not assess the relationship between IGDT-10 and different forms of functional impairment. We believe future studies would benefit from assessing gaming-related functional impairment using the 36-item WHODAS 2.0 and should consider using the clinician-administered version. Fourth, since there was a small number of extreme cases, EFA and CFA were not performed with independent subsamples, to avoid decreasing the power of the analysis. Finally, because of the cross-sectional design, we cannot infer causal relationships among the variables studied. Longitudinal studies may provide interesting information about the development and natural course of gaming-related functional impairment.

## Conclusion

In this study, we presented the psychometric properties of the Brazilian version of the IGDT-10 and explored the association between GD and functional impairment using network analysis. The IGDT-10 presented a unidimensional factor structure, with good internal consistency and satisfactory temporal stability. Participants who scored above the IGDT-10 cut-off point showed higher levels of functional impairment than those who scored below this threshold. Moreover, at the symptom level, the "negative consequences" criterion played a prominent role in the connection between IGDT-10 results and functional impairment. The IGDT-10 is a brief, easy-to-understand, valid, and reliable instrument, proving to be a suitable candidate for screening GD in future epidemiological studies in Brazil.
