# Supplementary material for: Validation of the Ten-Item Internet Gaming Disorder Test (IGDT-10) and its association with functional impairment in Brazilian gamers
Source: Trends Psychiatry Psychother. 2024 Nov 6;46:e20230622. doi: 10.47626/2237-6089-2023-0622 (PMC11815349; doi:10.47626/2237-6089-2023-0622)

http://doi.org/10.47626/2237-6089-2023-0622

Supplementary Material S1

Internet Gaming Disorder Test (IGDT-10)*

Por favor, leia as frases abaixo sobre jogos de videogame. O questionário se refere a jogos tanto online como offline, jogados em qualquer plataforma. Para simplificar, os termos "jogo" ou "jogar" são utilizados com esse mesmo significado.

Por favor, assinale na escala de 0 a 2 (0 = Nunca / 1 = Às vezes / 2 = Frequentemente) em que intensidade e frequência as frases abaixo se aplicaram a você ao longo dos últimos 12 meses.

	0	1	2
1. Quando você não estava jogando, com que frequência imaginou que estava jogando, ficou pensando em jogos anteriores ou em como seria o próximo jogo?			
2. Com que frequência você se sentiu inquieto, irritado, ansioso e/ou triste quando não pôde jogar ou quando jogou menos do que o habitual?			
3. Você sentiu necessidade de jogar com maior frequência ou jogou por mais tempo para ter a sensação de que tinha jogado o suficiente?			
4. Você tentou diminuir o tempo que passa jogando e não conseguiu?			
5. Você ficou jogando ao invés de encontrar seus amigos ou participar de atividades de lazer e passatempos que você costumava gostar?			
6. Você já jogou muito apesar de consequências negativas (por exemplo: dormir menos, não conseguir ir bem na escola ou no trabalho, discutir com familiares ou amigos e/ou negligenciar tarefas importantes)?			
7. Você já tentou esconder de sua família, amigos ou outras pessoas importantes para você o quanto você estava jogando, ou mentiu para eles sobre isso?			
8. Você já jogou para aliviar um sentimento negativo (por exemplo: desamparo, culpa ou ansiedade)?			
9. Você já arriscou ou perdeu algum relacionamento importante/significativo por causa do jogo?			
10. Você teve prejuízo no seu desempenho na escola ou no trabalho por causa do jogo?			

* Não é necessária autorização prévia para utilizar a "Ten-Item Internet Gaming Disorder Test". Apenas solicitamos que seja dado o devido crédito aos seus autores e que o presente artigo seja citado como referência.

http://doi.org/10.47626/2237-6089-2023-0622

Supplementary Material S2

Centrality analysis of network models

Figure S1 - Edge accuracy measures by the bootstrap method of the gaming disorder categorical diagnosis' network model

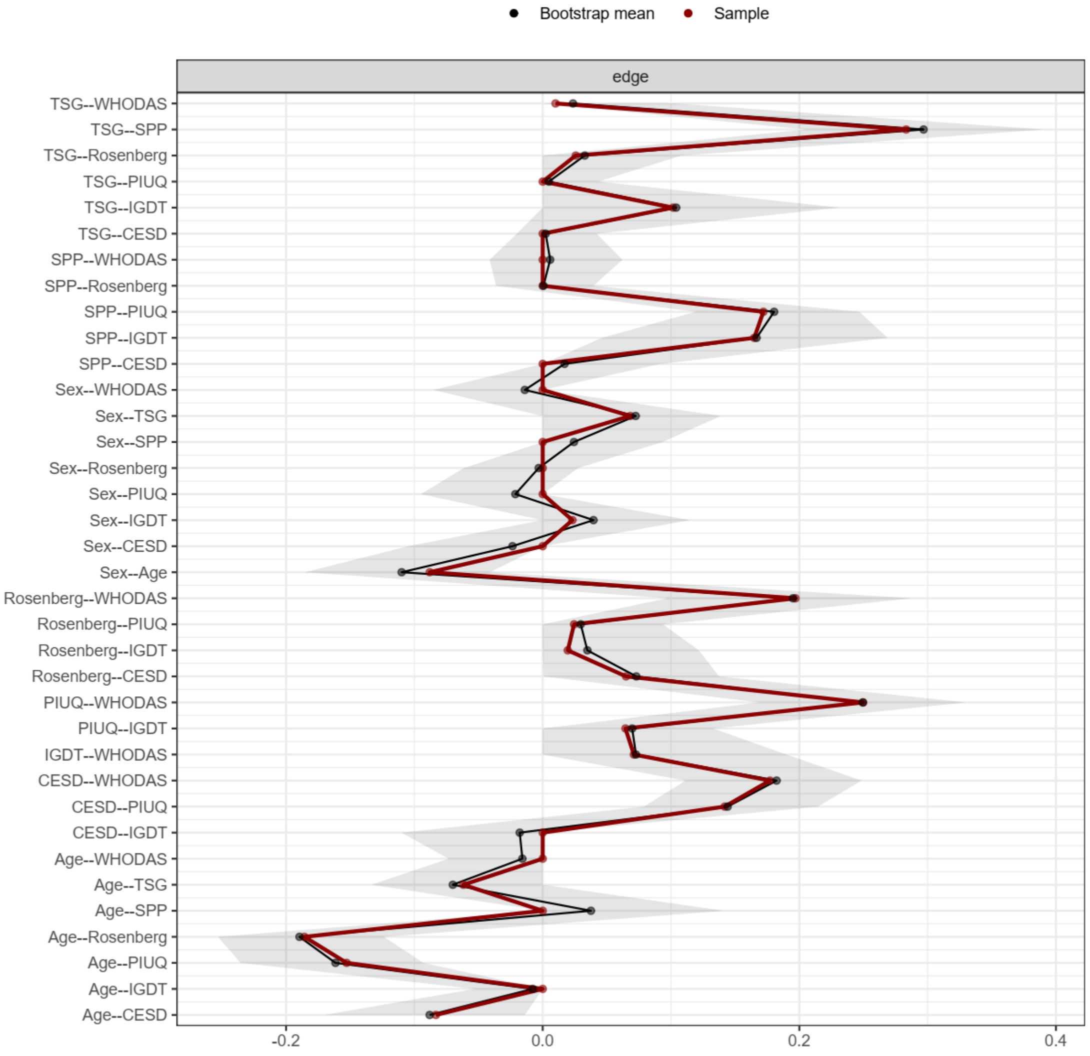

http://doi.org/10.47626/2237-6089-2023-0622

Figure S2 - Centrality accuracy measures by the bootstrap method of the gaming disorder categorical diagnosis' network model

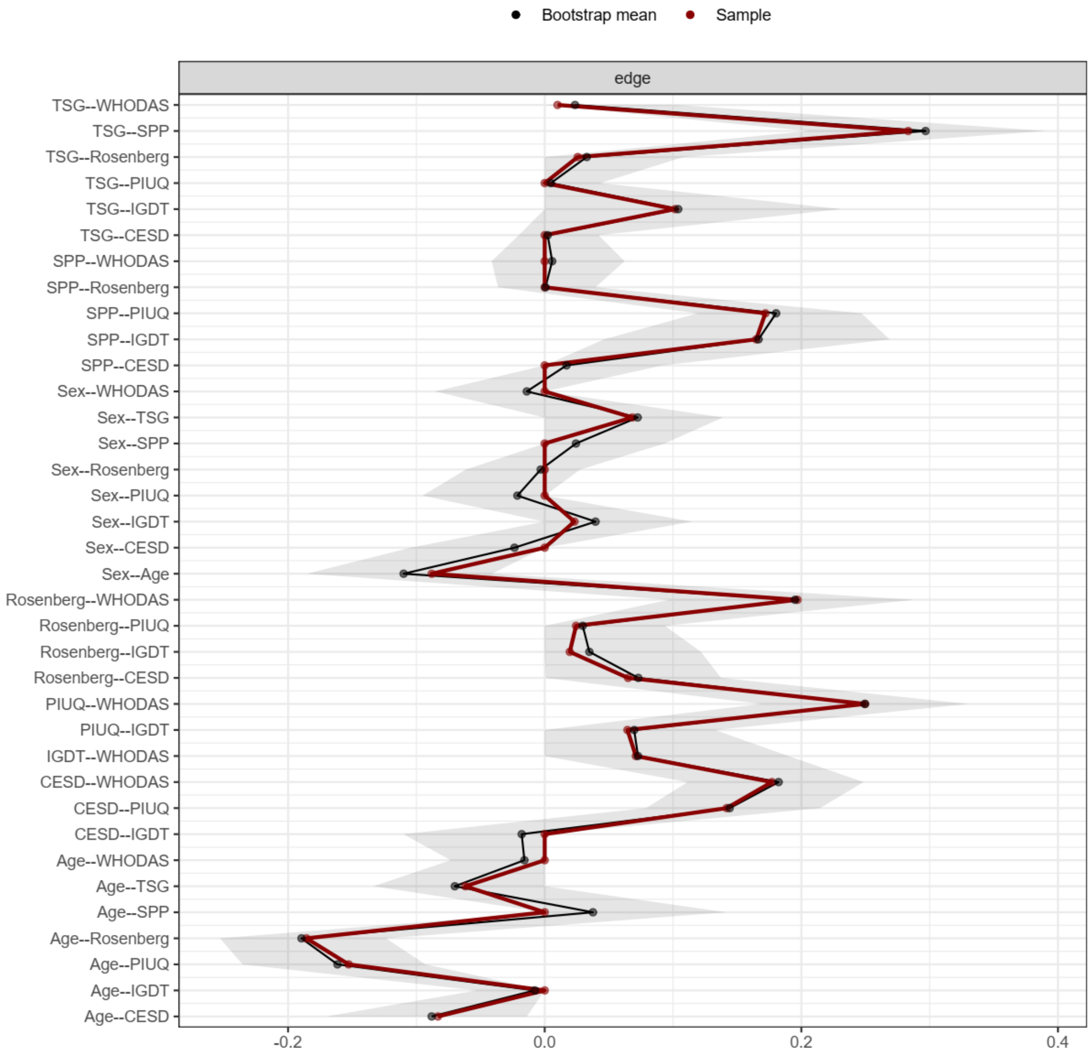

Figure S3 - Centrality stability measures by the bootstrap method of the gaming disorder categorical diagnosis' network model

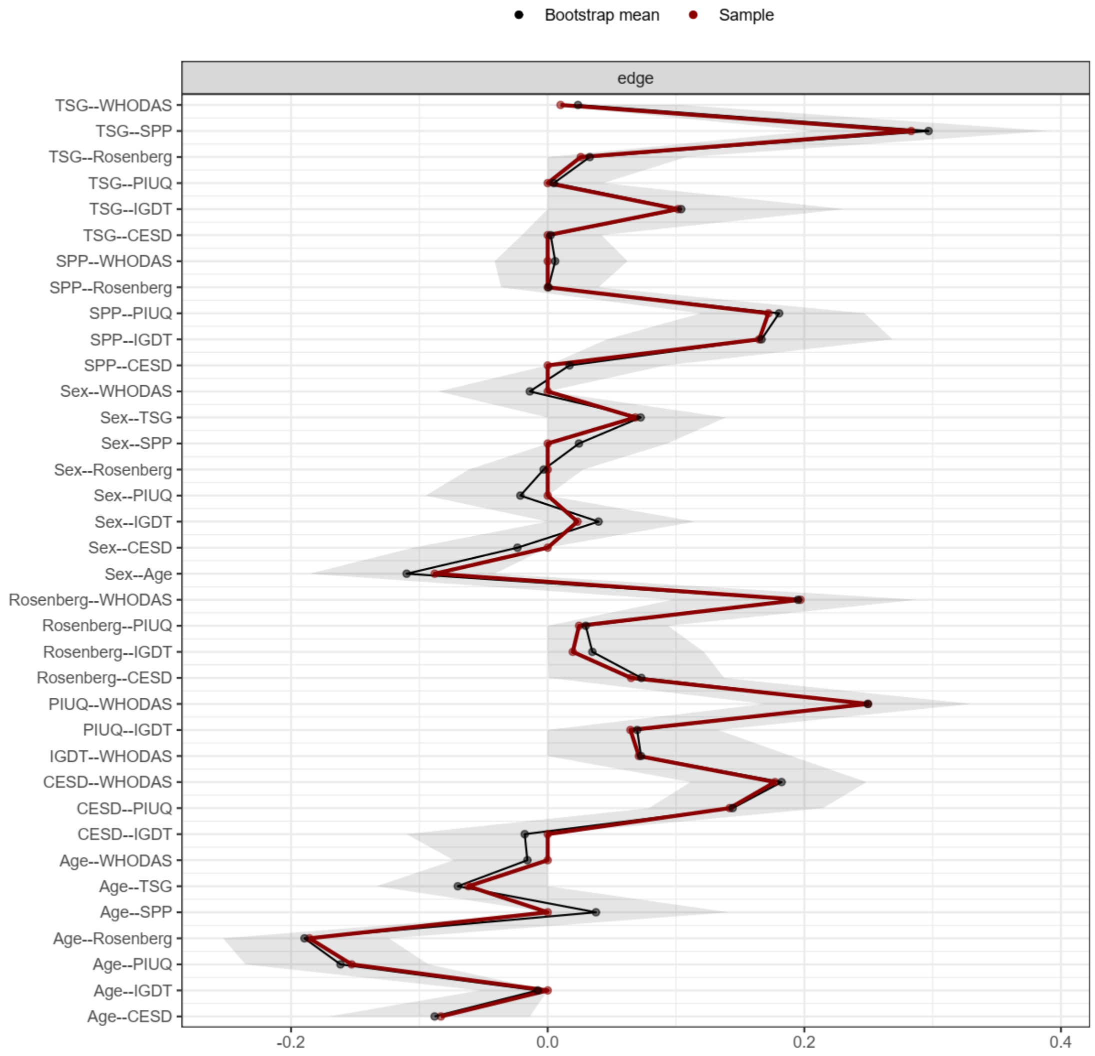

http://doi.org/10.47626/2237-6089-2023-0622

Figure S4 - Edge accuracy measures by the bootstrap method of the gaming disorder specific symptoms' network model

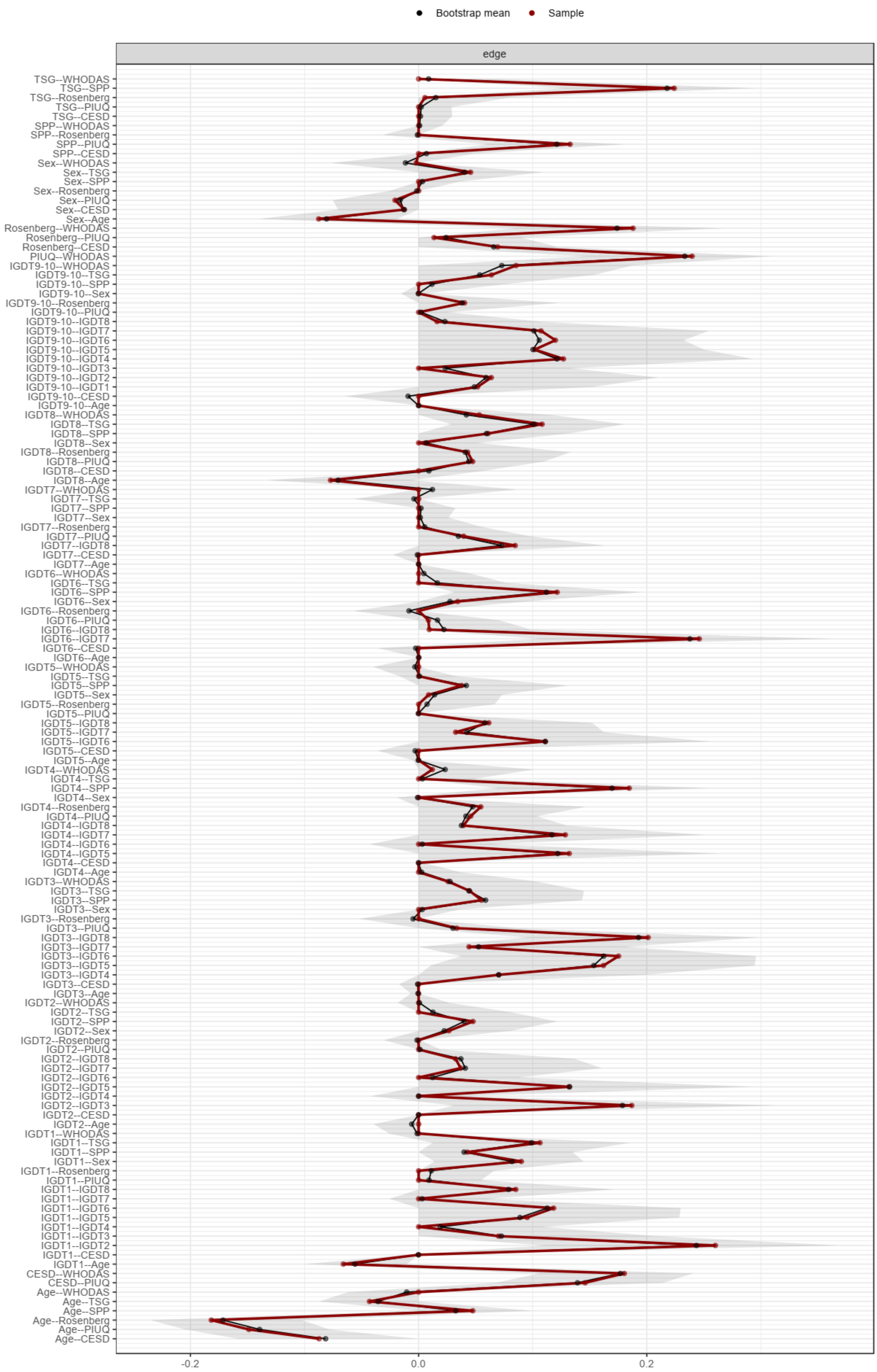

Figure S5 - Centrality accuracy measures by the bootstrap method of the gaming disorder specific symptoms' network model

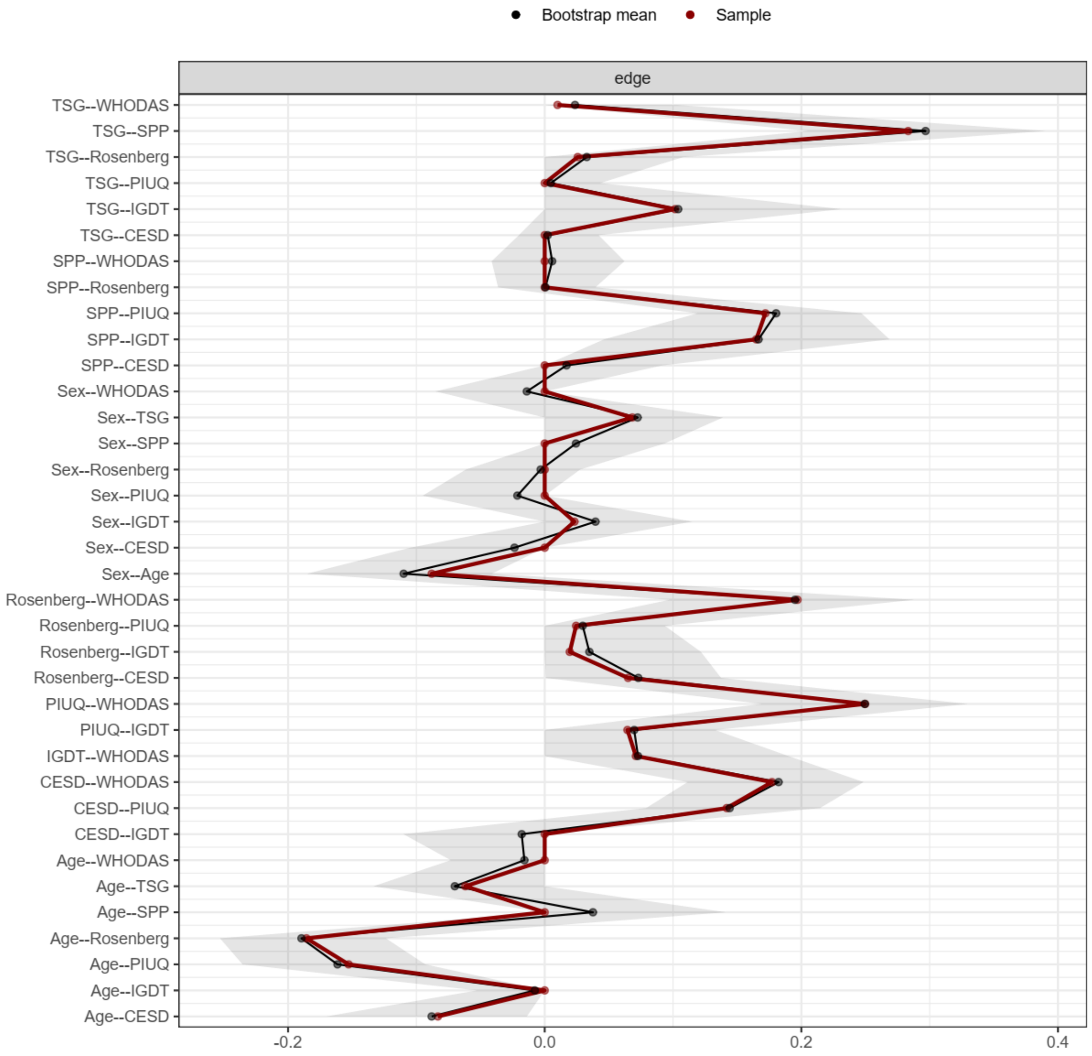

Figure S6 - Centrality stability measures by the bootstrap method of the gaming disorder specific symptoms' network model

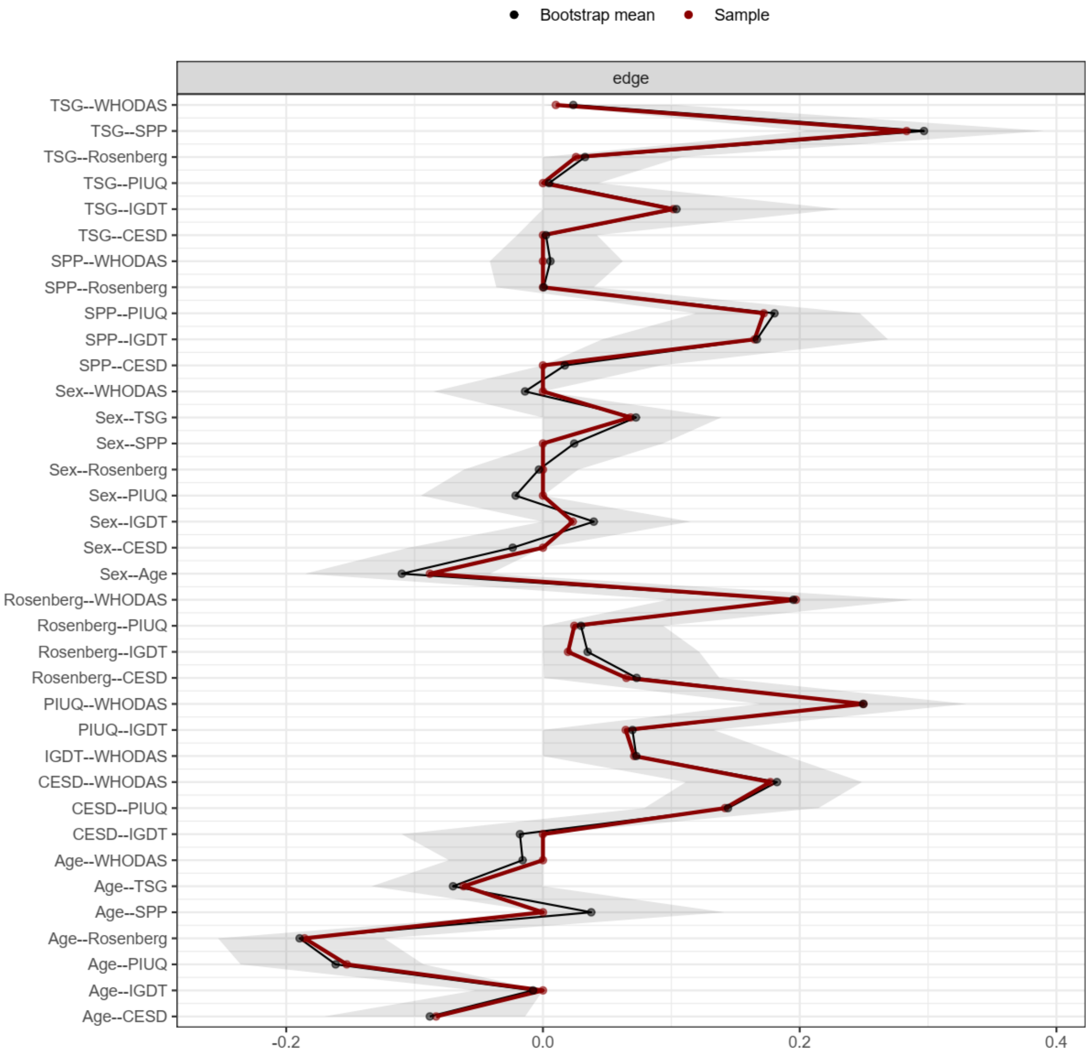

Supplement: Supplementary file 1 [file 2238-0019-trends-46-e20230622-suppl01.pdf]
